# Microdiversity of *Enterococcus faecalis* isolates in cases of infective endocarditis: selection of non-synonymous mutations and large deletions is associated with phenotypic modifications

**DOI:** 10.1080/22221751.2021.1924865

**Published:** 2021-05-21

**Authors:** G. Royer, L. Roisin, V. Demontant, S. Lo, L. Coutte, P. Lim, J. M. Pawlotsky, H. Jacquier, R. Lepeule, C. Rodriguez, P. L. Woerther

**Affiliations:** a Department of Microbiology, Henri Mondor Hospital, AP-HP, Université Paris-Est, Créteil, France; b LABGeM, Génomique Métabolique, Genoscope, Institut François Jacob, CEA, CNRS, Univ Evry, Université Paris-Saclay, Evry, France; c EA 7380, Université Paris-Est Créteil, Ecole nationale vétérinaire d’Alfort, USC Anses, Créteil, France; d Department of Cardiovascular Medicine and SOS Endocardites Unit, Henri-Mondor University Hospital, AP-HP, Créteil, France; e INSERM U955, Institut Mondor de Recherche Biomédicale, Créteil, France; f Bacteriology Unit, Lariboisière Hospital, APHP, Paris, France; g Université Paris Diderot, Sorbonne Paris Cité, UFR de Médecine, Paris, France

**Keywords:** *Enterococcus faecalis*, infective endocarditis, whole genome sequencing, microdiversity

## Abstract

*Context*: Today, infective endocarditis (IE) caused by *Enterococcus faecalis* represents 10% of all IE and is marked by its difficult management and the frequency of relapses. Although the precise reasons for that remain to be elucidated, the evolution of the culprit strain under selective pressure through microdiversification could be, at least in part, involved. *Material and methods*: To further study the *in situ* genetic microdiversity and its possible phenotypic manifestations in *E. faecalis* IE, we sequenced and compared multiple isolates from the valves, blood culture and joint fluid of five patients who underwent valvular surgery. Growth rate and early biofilm production of selected isolates were also compared. *Results*: By sequencing a total of 58 *E. faecalis* genomes, we detected a considerable genomic microdiversity, not only among strains from different anatomical origins, but also between isolates from the same studied cardiac valves. Interestingly, deletions of thousands of bases including the well-known virulence factors *ebpA/B/C*, and *srtC*, as well as other large prophage sequences containing genes coding for proteins implicated in platelet binding (PlbA and PlbB) were evidenced. The study of mutations helped unveil common patterns in genes related to the cell cycle as well as central metabolism, suggesting an evolutionary convergence in these isolates. As expected, such modifications were associated with a significant impact on the *in-vitro* phenotypic heterogeneity, growth, and early biofilm production. *Conclusion*: Genome modifications associated with phenotypic variations may allow bacterial adaptation to both antibiotic and immune selective pressures, and thus promote relapses.

## Introduction

Approximately 10% of infective endocarditis (IE) are caused by *Enterococcus faecalis*, which makes such infections difficult to manage and frequently relapse for reasons not entirely elucidated [1]. The genetic evolution of this bacterial strain, and its microdiversification under selective pressure, could play an important role in determining the infection outcome. This hypothesis has been supported by the identification of small colony variants (SCV) in culture [2–4] and by the observation of phenotypic or genetic changes associated with *E. faecalis* IE relapses [2,5]. However, until now, the phenotypic and genotypic diversity of *E. faecalis* has not been described nor extensively characterized over the course of IE. Such approaches were already proved relevant to understand the pathophysiology and to guide the therapy in other infectious diseases, such as osteoarticular infections or cystic fibrosis [6]. To determine the bacterial *in situ* genetic microdiversity and its relationship with IE phenotypic manifestations, we generated full-length sequences of *E. faecalis* isolated from valves, blood culture (BC), and joint fluid (JF) of five patients who had undergone valvular surgery.

## Materials and methods

### Patients and bacterial isolates

Five patients, who had valve replacement surgery following IE, were included. Socio-demographic and medical data were extracted from patients’ files. A single surgical specimen was taken from each valvular sample, shredded and seeded onto three different chocolate agar Petri dishes (PolyViteX, Biomerieux), respectively incubated in ambient air, 5% CO_2_ and anaerobic atmosphere for 48 h each. In order to catch as many diverse isolates as possible, three isolates (1 small, 1 medium and 1 large-sized) from each Petri dish were then subcultured and stored at −80°C for further analysis; i.e. a total of 9 isolates from each valvular sample. Whenever available, isolates taken from BC before surgery, as well as isolates from JF were also included and stored in the same conditions. Bacterial identification was performed on MALDI-TOF (Andromas, France), as described [7]. The samples were pre-extracted using bead beating on Tissue Lyser (Qiagen GmbH, Germany) and lysed with Proteinase K (Qiagen GmbH, Germany) at 56°C. Pre-lysates were extracted using DNA midi kit (QiaSymphony, Qiagen GmbH, Germany) according to manufacturer’s protocol. A negative environmental control (sterile water) sample and a positive bacteria-containing control (Microbial Community, ZymobioMics) were used in parallel. At the end of this step, DNA quality and quantity were tested using absorbance measuring and fluorescence by means of Quant-it kit on Varioskan (ThermoFischer Scientific Inc., France). Libraries were prepared using Nextera XT kit (Illumina, USA) and sequenced with NovaSeq 6000 S2 Reagent Kit v1.5 (300 cycles) on NovaSeq6000 (Illumina, USA) to obtain 2 × 150 bp paired-end reads and a coverage of at least 1000×. Libraries were validated if the negative control did not contain sequences of the bacteria under concern, and if all the bacteria of the positive control were detected.

### Sequence analysis

Genomes of each isolate were assembled using shovill 1.0.4 (https://github.com/tseemann/shovill), with SPAdes v3.13.1 [8], trimmomatic 0.39 [9], and Pilon 1.23 [10]. Contamination was checked using taxonomy workflow from CheckM v1.0.13 [11]. Only contigs > 500 bp were retained for further analyses.

We determined the Multi Locus Sequence Type (MLST) for each isolate based on PubMLST profile for *E. faecalis* (https://pubmlst.org/efaecalis/). The resistome, virulome, and plasmidome were analysed by means of blastN with ABRicate (https://github.com/tseemann/abricate) and Resfinder [12], VFDB [13], and PlasmidFinder [14] databases, respectively. Genomes were annotated on Prokka 1.13 [15] using standard parameters.

Microdiversity was assessed by Single Nucleotide Polymorphism/Insertion/Deletion (SNP-InDels) analysis, and relied on detecting large deletions. For a given patient, we compared all strains with each other using Breseq 0.31.1 with fold-coverage fixed at 100× [16]. Mutations with absolute coverage >10× and relative coverage ≥ 0.8 were further considered. SNPs detected as false positive upon comparing mapping reads of an isolate to the assembly of the same isolate, were discarded. Phaster was used to analyse sequences from prophages [17]. Clinker was used to illustrate specific deletions between isolates [18].

Finally, eggnog mapper 2.0.1 with emapperdb 5.0.0 [19] were used to determine the cluster of orthologous groups (COG) of non-synonymous mutated or deleted genes. Genome sequences and annotations are available under bioproject PRJEB36907.

### Minimum spanning trees

We created MLST-like profiles based on the presence/absence of SNP-InDel and large deletions as detected in the previous step. These profiles were used to draw Minimum Spanning Trees (MST) using Phyloviz [20]. Isolates were coloured according to the patient they were drawn from.

### Fitness assay

The Maximum Growth Rate (MGR) has been used as a proxy of the fitness [21]. Isolates were grown in Tryptic Soy Broth (TSB) supplemented with 1% glucose in deep-well plates for 18 h with shaking at 280 rpm at 37°C. Cultures were diluted 1:10,000 in a 96 flat-bottomed well plate containing 200 µL TSB with 1% glucose. Growth curves were determined at 37°C using a Tecan infinite 96-well plate reader, and the Maximum Growth Rate (MGR, in s^−1^) was determined as the maximum value of the derivative of logOD600, using R software, as previously described [21]. Experiment was performed in triplicate, from independent cultures.

### Early biofilm assay

Fresh colonies inoculated into Luria-Bertani (LB) were incubated overnight with shaking at 37°C. After centrifugation at 3000 rpm for 15 min, bacterial suspensions were adjusted to 3 McFarland (∼9 × 10^8^ CFU/mL) using a densitometer (Labomoderne, France). Further dilution in TSB with 1% glucose was necessary to obtain a working solution of 10^8^ CFU/mL. Inocula of 200 µL of bacterial suspension were spread on 96 flat-bottomed well plates (VWR, USA) to evaluate early biofilm production. A negative control sample containing a medium and two positive controls (B01: high and B02: low, both from ref. [5]) were added. After 6 h of static incubation at 37°C, plates were washed three times with Phosphate Buffered Saline (PBS) to remove unattached cells. Attached biomass was quantified by crystal violet (CV) staining, as previously described [22]. Briefly, 200 µL of CV solution (0.1%) was added to wells and then plates were incubated for 20 min at room temperature in the dark. Plates were washed three times with PBS, and CV was dissolved in acetic acid (30%). Absorbance at 550 nm was determined using a spectrophotometer (MultiskanTM FC, Thermo Fisher Scientific Inc., France). Experiment was run in sextuplicate and three independent experiments were performed.

### Statistical analysis

Growth rate and attached biomass data were analysed using JMP 14.0 software. These phenotypic assays were not performed for all isolates, particularly for the pairs of isolates for which a hypothesis could not be drawn based on genomic results. Statistical analysis was performed using Wilcoxon test for pairwise comparisons. *P*-values <0.05 were considered statistically significant.

## Results

From five patients, 58 full-length *E. faecalis* genomes were selected for sequencing based on their morphological criteria. The obtained 58 isolates correspond to 54 isolates from the six cardiac valves (9 isolates from each valve; Patient 5 had two valves removed), three isolates from BC (from Patient 2, Patient 3 and Patient 4) and one from JF (Patient 3 who experienced secondary localizations) (Tables 1 and S1). Phenotypic expression of the main genetic events was studied by means of fitness and early biofilm assays, using the closest isolates from the same patient as controls. All the results of these tests are presented and discussed below for each patient.

### Patient 1

An 82-year-old man was diagnosed in April 2018 with aortic IE caused by *E. faecalis*. He was initially treated with amoxicillin. At day 6, gentamicin was added since the routine antibiotic susceptibility test showed resistance to only erythromycin. Ten days after the first BC, he developed acute pulmonary oedema secondary to severe aortic regurgitation brought by a bulky aortic vegetation; hence the need for valve replacement.

Nine isolates (#1.1 to #1.9) from the aortic valve sample were studied. MLST analysis showed that they all belong to the sequence type (ST) 97. However, virulome analysis revealed the absence of *ebpA/B/C* and *srtC* in isolates #1.1 and #1.2 (Table 1). As far as the resistome was concerned, three isolates, including the 2 isolates lacking *ebpA/B/C* and *srtC*, also lacked two aminoglycoside resistance genes, *ant6*-Ia and *aph3′*-III, as well as *ermB*, which is involved in macrolide-lincosamide-streptogramin B resistance.

**Table 1.** Main characteristics of the isolates.

Patients	Isolates	Isolation source	MLST	Resistance genes*	Virulence genes*
Patient 1	1.1, 1.2	aortic valve	97	*lsaA, tetS*	EF0485, EF0818*, ace, bopD, cpsA, cpsB, efaA, fss1, fss2, fss3, gelE, prgB/asc10, sprE*
	1.5	aortic valve	97	*lsaA, tetS*	*ebpA, ebpB,ebpC, srtC,* EF0485, EF0818*, ace, bopD, cpsA, cpsB, efaA, fss1, fss2, fss3, gelE, prgB/asc10, sprE*
	1.3, 1.4, 1.6, 1.7, 1.8, 1.9	aortic valve	97	*ant6*-Ia , *aph3′*-III, *ermB, lsaA, tetS*	*ebpA, ebpB,ebpC, srtC,* EF0485, EF0818*, ace, bopD, cpsA, cpsB, efaA, fss1, fss2, fss3, gelE, prgB/asc10, sprE*
Patient 2	2.1 to 2.9	aortic valve (TAVI)	1002	*lsaA*	*ebpA, ebpB, ebpC, srtC,* EF0485, EF0818*, bopD, cpsA, cpsB, cpsC, cpsD, cpsE, cpsG, cpsH, cpsI, cpsJ, cpsK, efaA, fss1*
	2.10	bloodculture	1002	*lsaA*	*ebpA, ebpB, ebpC, srtC,* EF0485, EF0818*, bopD, cpsA, cpsB, cpsC, cpsD, cpsE, cpsG, cpsH, cpsI, cpsJ, cpsK, efaA, fss1*
Patient 3	3.1 to 3.9	mitral valve	206	*lsaA, tetM*	*ebpA, ebpB, ebpC, srtC,* EF0485*, bopD, cpsA, cpsB, cpsC, cpsD, cpsE, cpsF, cpsG, cpsH, cpsI, cpsJ, cpsK, efaA, fss1, fss2, prgB/asc10*
	3.10	bloodculture	206	*lsaA, tetM*	*ebpA, ebpB, ebpC, srtC,* EF0485*, bopD, cpsA, cpsB, cpsC, cpsD, cpsE, cpsF, cpsG, cpsH, cpsI, cpsJ, cpsK, efaA, fss1, fss2, prgB/asc10*
	3.11	joint fluid	206	*lsaA, tetM*	*ebpA, ebpB, ebpC, srtC,* EF0485*, bopD, cpsA, cpsB, cpsC, cpsD, cpsE, cpsF, cpsG, cpsH, cpsI, cpsJ, cpsK, efaA, fss1, fss2, prgB/asc10*
Patient 4	4.1 to 4.9	aortic valve	209	*lsaA*	*ebpA, ebpB, ebpC, srtC, bopD, cpsA, cpsB, cpsC, cpsD, cpsE, cpsG, cpsH, cpsI, cpsJ, cpsK, efaA, fss1, gelE, sprE*
	4.10	bloodculture	209	*lsaA*	*ebpA, ebpB, ebpC, srtC, bopD, cpsA, cpsB, cpsC, cpsD, cpsE, cpsG, cpsH, cpsI, cpsJ, cpsK, efaA, fss1, gelE, sprE*
Patient 5	5.1 to 5.9	mitral valve	55	*lsaA, tetM, tetS*	*ebpA, ebpB, ebpC, srtC,* EF0485, EF0818, *ace*, *bopD*, *cpsA*, *cpsB*, *efaA*, *fss1*
	5.10 to 5.18	tricuspid valve	55	*lsaA, tetM, tetS*	*ebpA, ebpB, ebpC, srtC,* EF0485, EF0818, *ace*, *bopD*, *cpsA*, *cpsB*, *efaA*, *fss1*

*Underlined genes are deleted at least in one strain.

**Figure 1.**
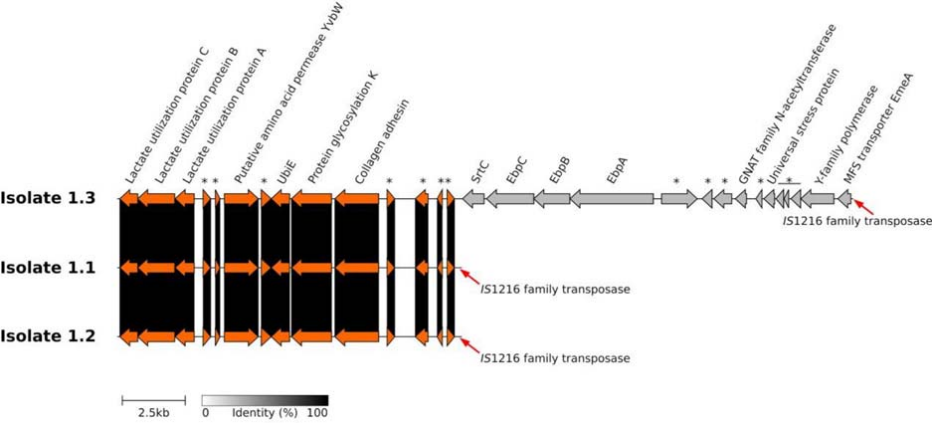
Genetic map of the 15,600 bp deletion containing *ebpA/B/C*, and *srtC* in patient 1 isolates #1.1 and #1.2. Names and/or locus tags of the predicted proteins are displayed. Asterisks stand for hypothetical proteins.

**Figure 2.**
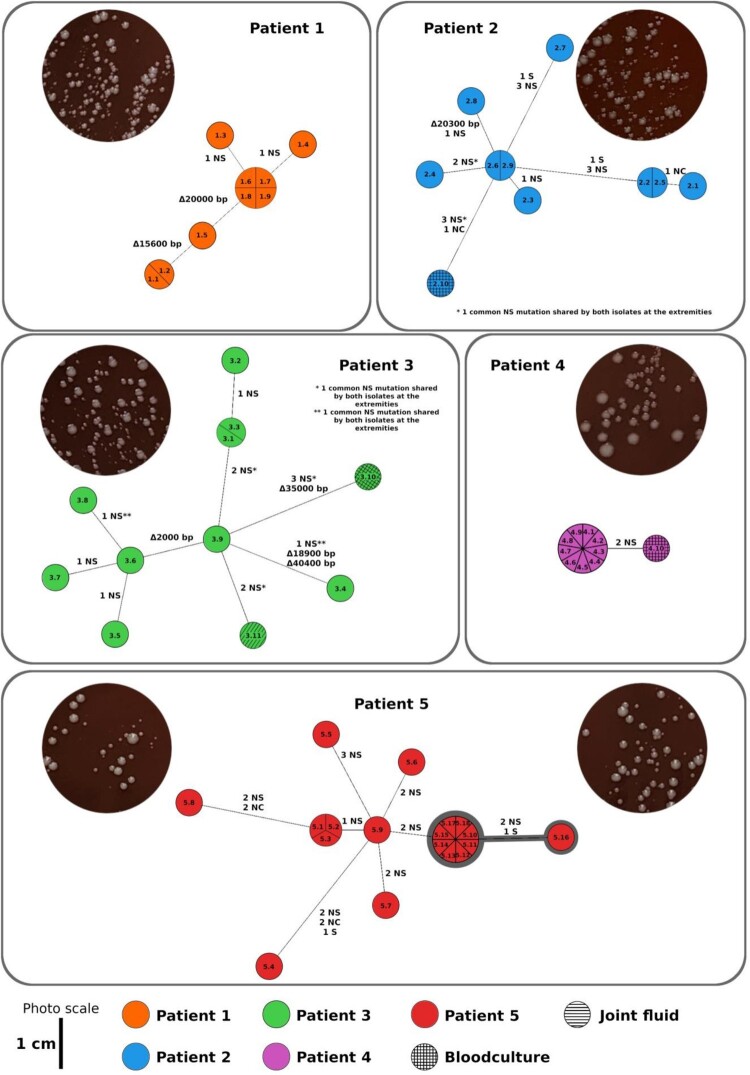
Minimum spanning trees of the patients’ isolates. Isolate identifiers are displayed in the coloured circles (e.g. 1.1, 1.2, 1.3, etc.). Non-synonymous (NS), synonymous (S), non-coding (NC) mutations and large deletions observed in isolates are represented by a solid line. In patient 5, isolates from the tricuspid valve are encircled in bold grey. Pictures of the colonies grown on PVX agar are shown for each valve.

We observed that a large sequence of 15,600 bp encompassing *ebpA*, *ebpB*, *ebpC*, and *srtC* was deleted within the genomes of isolates #1.1 and #1.2 (Figures 1 and 2). Analysis of isolates #1.1 and #1.2 sequences revealed the presence of *IS*1216 directly upstream of the deleted sequences. This *IS*1216 was also observed downstream of the 15,600 bp segment in the other isolates. A deletion, totalling nearly 20,000 bp, of multiple contigs of probable plasmid origin [blastN matches with pDEF-1 (accession number: CP031028) and pSULI (accession number: CP043327.1)], involving the fore-mentioned resistance genes (*ant6*-Ia, *aph3′*-III, and *ermB*), was also detected in the sequence of these two isolates and of a third one (#1.5) (Figures 2 and S1). As the entire contigs were deleted from the three isolates, we were not able to analyse the flanking regions. Finally, only two SNP-InDels were observed in two isolates: a 30-bp deletion in *tmpC* gene which codes for membrane lipoprotein TmpC in one of them, and a non-synonymous mutation in *fba* gene which codes for fructose-bisphosphate aldolase in the other one (Figure 2).

**Figure 3.**
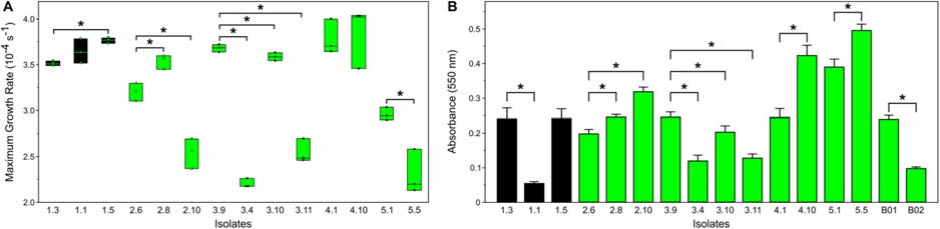
Phenotypic assays performed on a selection of isolates. Isolate #1.3, #3.9, #4.1, #2.6 and #5.1 are used as controls for patients 1, 2, 3, 4, and 5, respectively. (A) Maximum Growth Rate (MGR, in s−1) and (B) early biofilm production of the *E. faecalis* isolates. *p < 0.05.

For phenotypic assessment, we compared the isolate lacking *ebpA/B/C/srtC* virulence factors (#1.1) and the isolate lacking only the plasmidic sequences (#1.5) with their closest isolate (#1.3) (Figure 2). Isolate #1.1 was characterized by a significant decrease of early biofilm production (*p* < 0.0001) without growth defect (*p* = 0.1266) (Figures 3 and S2). Interestingly, this decrease of early biofilm production associated with the loss of *ebpA/B/C/srtC* virulence factors, observed in isolates #1.3 and #1.1, was comparable with that of the controls B01 and B02, which were distinguished from each other by the loss of the same virulence factors. Conversely, the isolate lacking plasmidic sequences (#1.5) showed increased growth with unchanged early biofilm production.

### Patient 2

An 80-year-old woman had a pacemaker installed in 2013 and underwent Transcatheter Aortic Valve Implantation (TAVI) in 2017. In January 2018, fully-susceptible *E. faecalis* was detected in her BC, and thus was diagnosed with aortic IE and probable spondylodiscitis. Three days after the first positive BC, amoxicillin and gentamicin were prescribed for one day and then switched to amoxicillin plus ceftriaxone. On day 14, the infected aortic valve implant was removed.

Nine valvular isolates (#2.1 to #2.9) and one BC isolate (#2.10) were analysed. All of them belonged to ST1002. The 10 isolates from this patient showed the same resistome and virulome findings (Table 1) and shared several genetic characteristics. Precisely, 13 non-synonymous mutations were found in genes involved in sugar and amino-acid transport, phosphoenolpyruvate-protein phosphotransferase, glutamate 5-kinase, and polyribonucleotide nucleotidyltransferase. Two synonymous mutations were also observed, as well as two other mutations in non-coding regions (Figure 2 and Table S2). Moreover, isolate #2.8 lacked approximately 20,300-bp sequence carrying many mobile genetic elements, such as insertion sequences and other genes that code for lysophospholipase, TetR/AcrR family transcriptional regulator, and copper oxidase (Figure S3). As all the contigs were deleted from isolate #2.8, we were not able to analyse the flanking regions.

Given the high level of genetic diversity observed in the studied isolates from patient 2, phenotypic assays were performed on isolates #2.6 and #2.8, where only the latter showed the 20,300-bp deletion, and on isolate #2.10 obtained from the BC. Despite the absence of any pertinent genetic hypothesis, isolate #2.10 displayed more early biofilm production and lower growth ability.

### Patient 3

A 73-year-old woman had susceptible *E. faecalis* IE developed on a bioprosthetic mitral valve and complicated with secondary hip septic infection in August 2018. She was first put on vancomycin and gentamicin and then switched to amoxicillin plus gentamicin after microbiological testing. Given the high risk of embolism, the patient underwent surgery nine days after the initial BC.

Nine isolates from the mitral valve (#3.1 to #3.9), as well as two isolates from the BC (#3.10) and the JF (#3.11) were studied. All of them belonged to ST206 and had identical resistome and virulome (Table 1).

**Figure 4.**
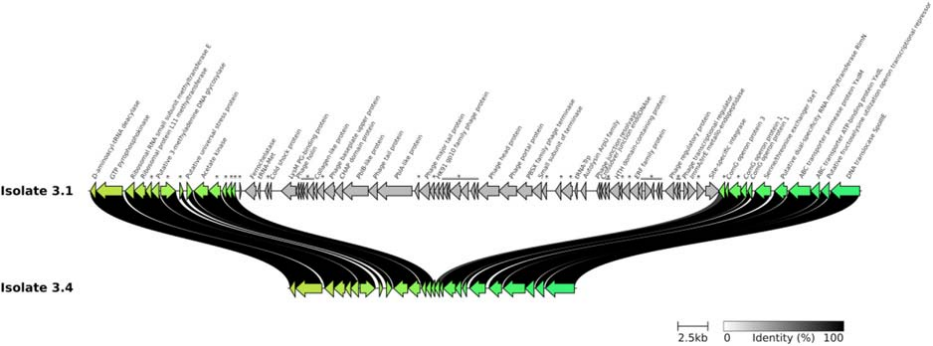
Genetic map of the 40,400 bp deletion corresponding to a prophage sequence in patient 3 isolate #3.4. Names and/or locus tags of the predicted proteins are displayed. Asterisks stand for hypothetical proteins.

Three large and one small deletions were found in the 11 isolates. Two large deletions of 40,400 bp and 18,900 bp were found in isolate #3.4. The 40,400 bp deletion corresponded to phage pp4, which was deleted from *E. faecalis* V583 (region EF1988 to EF2043, genome accession number AE016830.1) and replaced by an *attB* site (TTTGCCACTCCCCATCTGAAATT) (Figure 4). Interestingly, phage pp4 carried two genes that code for PblA and PblB proteins, which promote adhesion to human platelets [23]. The deleted 18,900 bp sequence carried genes coding for proteins involved in sugar transport, RND and MFS transporters, and other proteins (Figure S4). The small deletion of 2000 bp was found in four isolates (#3.5, #3.6, #3.7, and #3.8) and corresponded to a small portion of phage pp4. This deleted sequence included two genes coding for hypothetical proteins, tRNA-Trp (EF_tRNATrp2 from *E. faecalis* V583), and a gene coding for a transcriptional regulator that belongs to ArpU family (EF_2024 *E. faecalis* V583). The BC isolate displayed the third large deletion of 35,000 bp which corresponded to another prophage, identical to the one found in *E. faecalis* strain symbioflor (HF558530.1) (Figure S5). Phaster analysis revealed the proximity of this phage to *Enterococcus* phage vB_EfaS_IME197 (accession number: NC_028671.2).

Upon comparing all the isolates together, they differed by at least one non-synonymous mutation, except isolates #3.1 and #3.3 which were identical. These mutations concerned genes coding for ribosomal proteins, ADP-ribose pyrophosphatase, PTS system, ribonuclease, transcriptional regulators, and translation initiation factors.

The phenotypic analysis of this case covered the isolate which missed pp4 phage sequences (#3.4) and both isolates from BC and JF (#3.10 and #3.11). The closest isolate (#3.9) was used as a control (Figure 2). Growth rate and early biofilm production of the three tested isolates were significantly reduced, although the BC isolate was less affected (Figure 3).

### Patient 4

A 76-year-old man was diagnosed with susceptible *E. faecalis* IE of the aortic valve in February 2018. One day after having a positive BC test, the man was put on amoxicillin and gentamicin, and then switched to amoxicillin plus ceftriaxone following microbiological results. The patient was operated on two days after because of severe aortic regurgitation.

Nine valvular isolates (#4.1 to #4.9) and one isolate from the initial BC (#4.10) were taken and all belonged to ST209 and showed identical resistome and virulome.

Only two non-synonymous mutations were found in the BC isolate as compared with the valvular isolates. They were located in a region coding for manganese ABC transporter substrate-binding lipoprotein and lipopolysaccharide biosynthesis protein (Table S2). The poor molecular diversity of this case fitted the poor macroscopic diversity, which was characterized by the absence of SCV as shown in the Petri dish picture (Figure 2).

Upon phenotypically comparing the BC isolate (#4.10) with one of the valvular isolates (#4.1), the former showed unchanged growth rate (*p* = 0.5127) and a significant increase in early biofilm production (*p* < 0.0001) (Figure 3).

### Patient 5

A 65-year-old man was diagnosed in March 2018 with mitral and tricuspid IE caused by a susceptible *E. faecalis* strain. The progressive development of spondylodiscitis and knee arthritis suggested a long-lasting infection. The patient was treated with amoxicillin plus gentamicin from the day following the first positive BC and subjected two days later to surgical replacement of both valves since the echocardiography showed a 15 mm vegetation.

Nine isolates from each valve (#5.1 to #5.9 and #5.10 to #5.18, respectively) were analysed. All of them belonged to ST55 and had identical resistome and virulome.

Interestingly, half of the mitral valve isolates differed by at least two non-synonymous mutations (Figure 2), whereas nearly all of the tricuspid valve isolates were identical. Only two non-synonymous mutations were found in the nine isolates from the tricuspid valve, suggesting a two-step evolution process (Table S2).

Among the 22 detected SNP-InDels, those involving genes coding for proteins acting in central metabolism, e.g. *proB*, *fba*, *pfkA*, and *cfiB*, were non-synonymously mutated in several isolates (Table S2). One isolate (#5.5) carried a non-synonymous mutation in a gene coding for WxL domain-containing protein, which exhibited 56% protein identity and 100% coverage with EF_3248 from *E. faecalis* V583. Such proteins were described as cell wall binding proteins [24] and could play a role in IE pathogenesis [25].

The isolate carrying the non-synonymous mutation in the gene coding for WxL protein (#5.5) was compared with one of the three identical isolates from the mitral valve (#5.1). Isolate #5.5 showed a reduced growth rate and higher early biofilm production than the control (Figure 3). This improvement could also be accounted for the three non-synonymous mutations found in the control in *greA* (Transcription elongation factor GreA) and *pfkA* (ATP-dependent 6-phosphofructokinase). In absence of available literature on this subject, more data are needed to conclude on the potential role of each of these elements.

## Cluster of orthologous groups classification analysis

**Figure 5.**
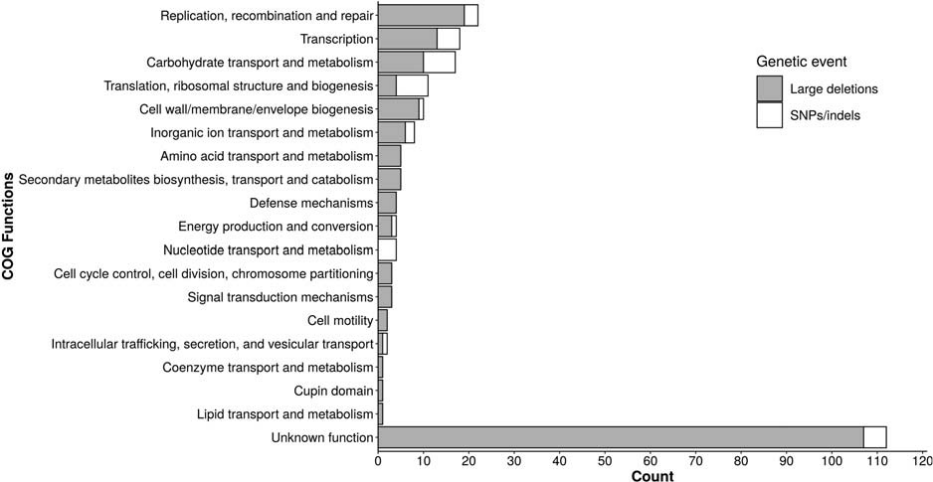
Cluster of orthologous groups functions of the genes affected by deletions or non-synonymous mutations among the 58 *E. faecalis* genomes.

To obtain a more generalized insight into the microdiversity of *E. faecalis*, we analysed non-synonymous SNP-InDels and large deletions in terms of Cluster of Orthologous Groups (COG) classification. Given the probable phage or plasmid origin of many of the missing genes and their erroneous annotations in available databases, many genes remained unclassified or were put under unknown functions (Figure 5 and Table S3). However, genes in which SNP-InDels were evidenced – and to a lesser extent those missing as a result of large deletions – were more often involved in cell cycle functions (e.g. replication, recombination and repair, transcription, translation, and ribosomal structure and biogenesis), as well as in central metabolism (e.g. carbohydrate transport and metabolism). Cell wall, membrane, and envelope biogenesis were mainly affected by large deletions, such as the one observed in patient 1 with *ebp* operon. Overall, these modifications highlight a positive selective pressure involving mainly important functions related to the cell cycle and central metabolism.

## Discussion

Overall, the diversity observed was high at all of the explored levels in isolates sampled from excised valvular specimens, BC, and secondary localizations in patients with IE. Isolates belonged to different MLST, namely 97, 1002, 206, 209, and 55. Acquired resistance genes were different from one strain to another. Finally, a high diversity was also evidenced regarding the virulence genes, since only *ebpA, ebpB, ebpC*, and *srtC*, which are of particular interest in IE and belong to the same operon, were common to all strains. This diversity, already observed in other chronic infections or prosthesis-related infections [6], is brought by the strong selective pressure incurred by the host immune response and the administered antibiotics. As a result, the bacteria that lost certain proteins targeted by the immune system or acquired certain SNPs in genes involved in the regulation of replication, transcription, or metabolism to help it resist better, might be positively selected. This hypothesis is reinforced by the phenotypic modifications observed upon comparing the adhesion or the growth rates of isolates from each patient.

The presence of SCV was marked in four of the five studied patients. This phenotypic heterogeneity matched the high genotypic diversity we observed in this study. However, no relation between the size of the colonies and the corresponding growth rates was observed. In patient 4, few SCVs were found and the genome comparison failed to show any significant difference between isolates taken from the valve. This patient was operated on early, suggesting that the short infection duration limited the bacterial diversification within the valve. In contrast, patients 2, 3, and 5, who developed valve prosthesis infection or secondary infections, had longer evolution of their IE and, as a result, exhibited greater genetic diversity in isolates. In patient 5, the genetic diversity of the strain probably reflected the temporal evolution of the infection since all of the tricuspid valve isolates carried two specific SNPs, as compared with the mitral isolates.

The first large deletion included VFs *ebpA/B/C*, and *srtC* [24] and was detected in two isolates from patient 1. We had already observed such a large deletion in a case of *E. faecalis* IE relapse, thus we used its isolates B01 and B02 [5] as controls in our early biofilm assay. An interesting parallelism was observed there, which suggests a decrease in early biofilm production in isolates exhibiting this deletion. This could be explained by the immune pressure, as previously hypothesized. Given that Ebp factors are the key targets of the immune response [26], their loss could help escape host defences, thereby favouring relapse. However, the major drawback of this “strategy” is its significant impact on bacterial metabolism, especially biofilm formation [5].

The first deletion found in patient 3 corresponded to pp4 prophage [23], which carries genes coding for PlbA and PlbB proteins involved in platelet binding. By analogy, pp4 loss may decrease the host/bacteria interaction, thereby help escape the immune system. Of note, pp4 sequence deletion is translated into a decrease of both growth and early biofilm production. The other deletion detected in the BC isolate of patient 3 also corresponded to a prophage sequence, but the phenotypic consequences of this loss were less marked and the immunological impact was hypothetical. Altogether, these findings underline the probable role of phages in modifying genome plasticity of bacteria under strong selective pressure.

A few other deletions involved sequences of probable plasmid origin, as shown by blastN analysis on the NCBI website, and some contained resistance genes, such as in patient 1 (isolates #1.1, #1.2, and #1.5). The study of genomic mutations revealed common patterns in several patients. For instance, *fba* (fructose biphosphate aldolase) was mutated in patients 1 and 5, *sorA* (PTS system, mannose-specific IIC component) in patients 2 and 3, *proB* (glutamate 5 kinase) in patients 2 and 5, and *nudF* (NUDIX hydrolase) in patients 3 and 5. Even if the observed mutations were different, their presence in a few specific genes probably points out an evolutionary convergence in these isolates.

In this observational study, the genetic and phenotypic comparisons were performed on a limited number of isolates, which were chosen according to the patients they were isolated from and to their genetic distance. Although the tools used to assess both the growth rate and the biofilm production do not reproduce the very particular *in vivo* conditions observed in the valvular micro-environment, we were able to observe a clear relationship between genome modifications and changes in phenotypic manifestations. However, linking growth rate or early biofilm production variations to a single mutation or deletion was impossible, since these parameters resulted from the overall impact of a sum of distinct genetic events. Additionally, we were not able to appraise the whole spectrum of the bacterial diversity not only because the culture conditions and the number of selected isolates were limited, but also because the isolates sharing identical or closely related genomes were not phenotypically compared. Finally, to better identify the role of the identified mutations and their link with the observed phenotypic modifications, the pairs of isolates that only differ by the mutation/deletion of interest should be studied through directed mutagenesis and testing in *in-vitro* or in animal IE models.

Of course, in addition to the selective pressure exerted by the patient’s immune response, antibiotics may have played a role in the selection of certain SNP or deletions. However, because our cohort was conducted on only five patients and the antibiotics therapies (course, doses, combination), as well as the pattern of infection evolution differed from one case to the other, it was impossible to make any hypothesis regarding the impact of one particular antibiotic regimen on the selection of all SNPs or deletions we detected.

In conclusion, by sequencing a total of 58 full-length *E. faecalis* genomes, this study highlighted a considerable genomic microdiversity, not only among strains from different anatomical origins, but also between isolates from the same cardiac valves. Interestingly, the observed diversity seemed to correlate with the duration of the infection since the number of mutations was higher in patients with suspected long-lasting infections. This work also showed the rate of large deletions including the one that encompassed *ebpA, ebpB, ebpC*, and *srtC* genes and could play a pivotal role in escaping the host immune response, as previously described in the case of relapse. Large deletions, e.g. of prophage sequences, as well as mutations affecting key functions could also contribute to the survival of bacteria under intense selective pressure; nonetheless, experimental studies are needed to understand the specific impact of each of them.

## Supplementary Material

Figure_S5.jpeg

Figure_S4.jpeg

Table_S3_COG.xlsx

Table_S2_mutations.xlsx

Table_S1_culture_conditions.xlsx
