# Supplementary figures and images for: Microdiversity of Enterococcus faecalis isolates in cases of infective endocarditis: selection of non-synonymous mutations and large deletions is associated with phenotypic modifications
Source: Emerg Microbes Infect. 2021 May 21;10(1):929–38. doi: 10.1080/22221751.2021.1924865 (PMC8158287; doi:10.1080/22221751.2021.1924865)

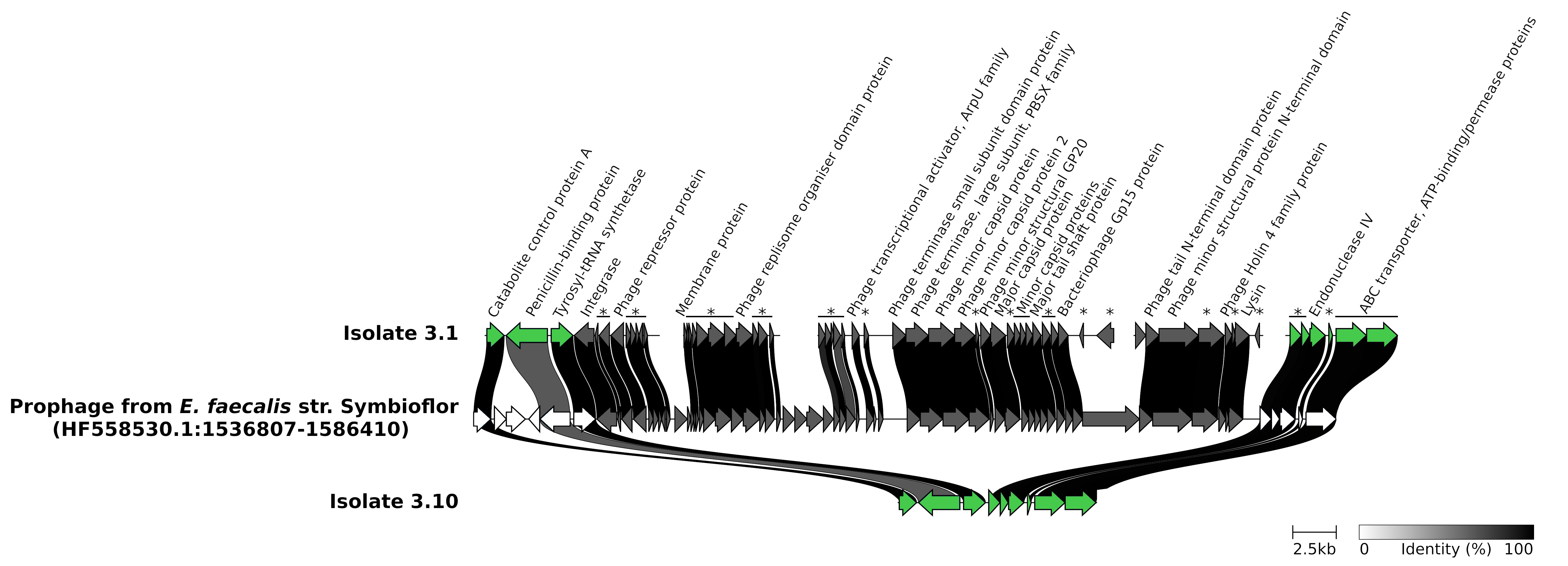

Supplement: Figure_S5.jpeg [file TEMI_A_1924865_SM1474.jpeg]

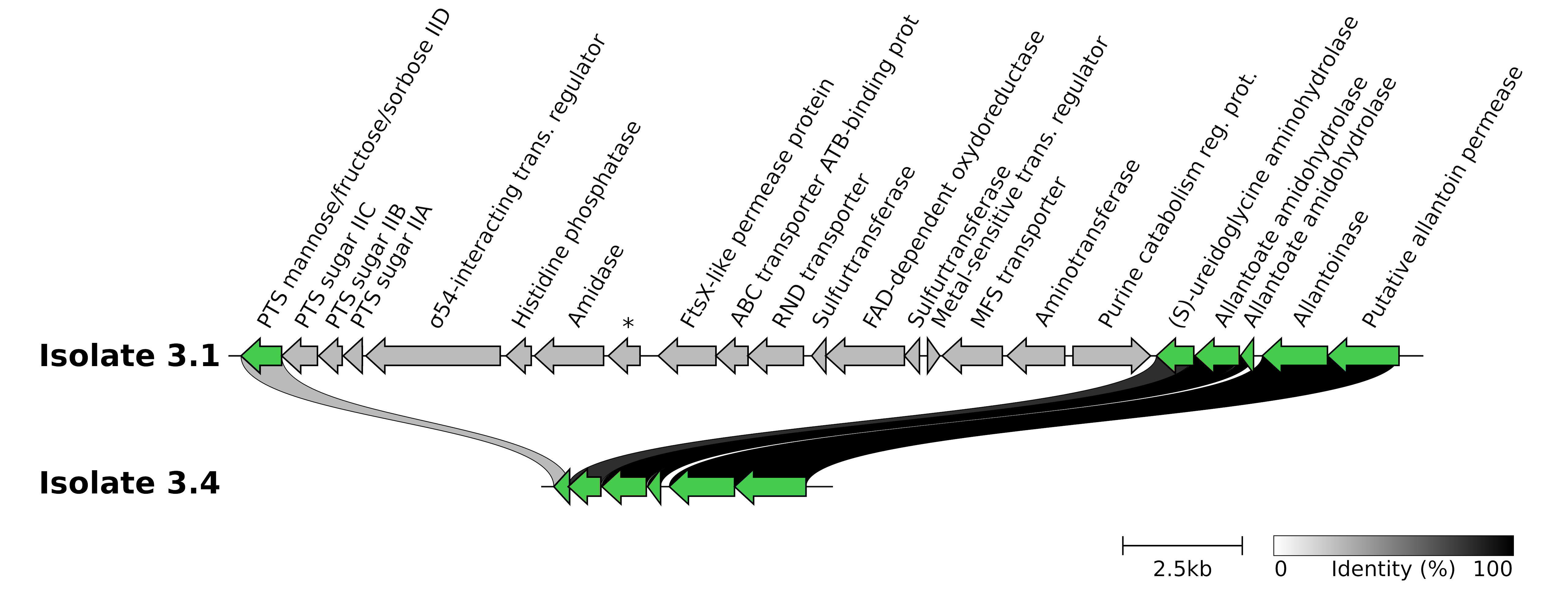

Supplement: Figure_S4.jpeg [file TEMI_A_1924865_SM1473.jpeg]
